# Surgical Outcome of Complex Knee Deformity Correction in a Girl With Ellis-van Creveld Syndrome: A Report of a Rare Case

**DOI:** 10.7759/cureus.33863

**Published:** 2023-01-17

**Authors:** Vismay V Harkare, Sushil Mankar, Mohammed Faizan, Amit Nemade

**Affiliations:** 1 Orthopedics and Traumatology, N.K.P Salve Institute of Medical Sciences and Research Center, Nagpur, IND

**Keywords:** soft tissue release, case report, chondroectodermal dysplasia, ellis van creveld syndrome, severe genu valgum

## Abstract

Ellis-van Creveld (EVC) syndrome is a rare inherited condition with inheritance, which is autosomal recessive in nature and is also described as skeletal dysplasia (chondroectodermal). The patients present with a grave genu valgum deformity which is a major challenge in orthopedics. The current case report presents a young girl of the juvenile age group who came with deformity over the bilateral lower limb with difficulty in walking and bilateral upper limb polydactyly. The patient underwent relevant investigations and examinations which were suggestive of bilateral genu valgum deformity. Since the deformity was significant, a corrective osteotomy with soft tissue release was planned followed by rehabilitative physiotherapy. Considering the extensive nature of the surgery, a staged procedure was planned. The patient on follow-up presented with a good range of motion and improved gait. Various treatment modalities have been described for the correction of the deformity but few of them are done in patients with EVC syndrome and they state varying results and high incidences of relapse. The present study focuses on corrective osteotomy with soft tissue release as a treatment modality and describes the outcome associated with the modality. Thus, stating that with proper planning and postoperative rehabilitation the patient can achieve a good functional outcome.

## Introduction

Ellis-van Creveld (EVC) syndrome is an extremely uncommon type of dysplasia that was first mentioned by Van Creveld and Ellis in 1940. The prevalence is estimated to be around 0.1/million population [1]. Particularly affected are the Pennsylvania Amish, in whom the condition occurs in one in 5000 births. It is due to a mutation in the evc/evc 2 gene and has an autosomal recessive inheritance. It is characterized by four components - polydactyly, congenital heart defects, ectodermal dysplasia affecting the nails, teeth, and hair, and chondrodysplasia [1,2].

These patients present with a grave angular deformity of the knee that is genu valgum, which is a surgical challenge for orthopedic surgeons. It is due to the involvement of the lateral tibial plateau which is affected by the primary genetic-based dysplasia and the surrounding soft tissue contractures that force the tibia into valgus deformity [3]. A profound “Saucer-like” crater of the lateral portion of the tibial articular surface and profound valgus of the proximal tibia is the chondro-osseous deformity seen. The deformity consists of dislocation or lateral patellar subluxation along with severe contractures of the joint capsule, vastus lateralis, and iliotibial band [3].

The patient also presents with acromelic and mesomelic shortening of the limb. There is marked shortening in the distal aspect of the limb (e.g. phalanges). The hallmark of the syndrome is polydactyly which is often postaxial [1,2]. Fifty to sixty percent of patients come with cardiac anomalies, most likely a single atrium (40%) [1,2].

## Case presentation

Patient information

A young girl of 11 years of age with short stature was brought for consultation in the orthopedic outpatient clinic for severe deformities of the knee and difficulty in walking. The patient had been operated on for a single atrium at an early age.

Clinical findings

On examination, she had short stature, deformed teeth, acromesomelic shortening, postaxial polydactyly, severe genu valgum, and bilateral dislocated patellae. The patient had walking difficulty and a waddling gait.

Timeline

The patient presented with complaints of progressive deformity in both knees and walking difficulty at a young age. The deformity was first noticed by the patient’s relatives when she was 1.5 years old and soon after she started walking. The deformity was slowly progressive and the patient was taken to an orthopedic surgeon for management. But in view of the repeated episodes of congestive heart failure, management for the same was deemed a priority and the patient was operated on for a single atrium at 2.5 years of age. The patient was first managed for deformity of the knee at 10 years of age after her cardiac condition was stable. The first procedure (bilateral medial proximal tibia and distal femur epiphysiodesis with staples along with bilateral supracondylar femoral osteotomy) was done at some other center and did not have satisfactory results, hence a revision surgery was planned. The patient was followed up for physiotherapy and had an excellent outcome with no signs of recurrence at the five-year follow-up.

Diagnostic assessment and interpretation

**Figure 1:**
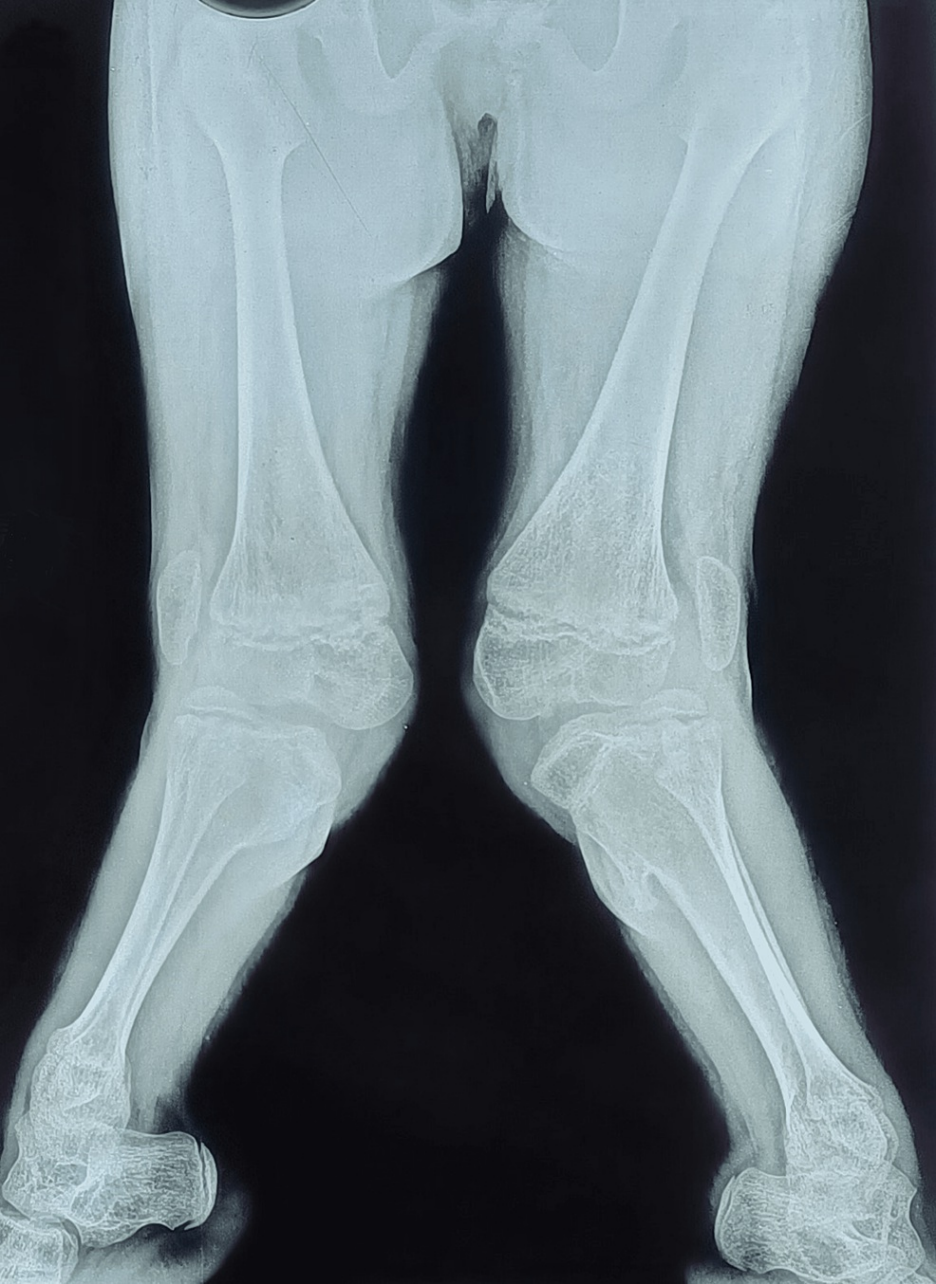
Severe bilateral genu valgum deformity.

**Figure 2:**
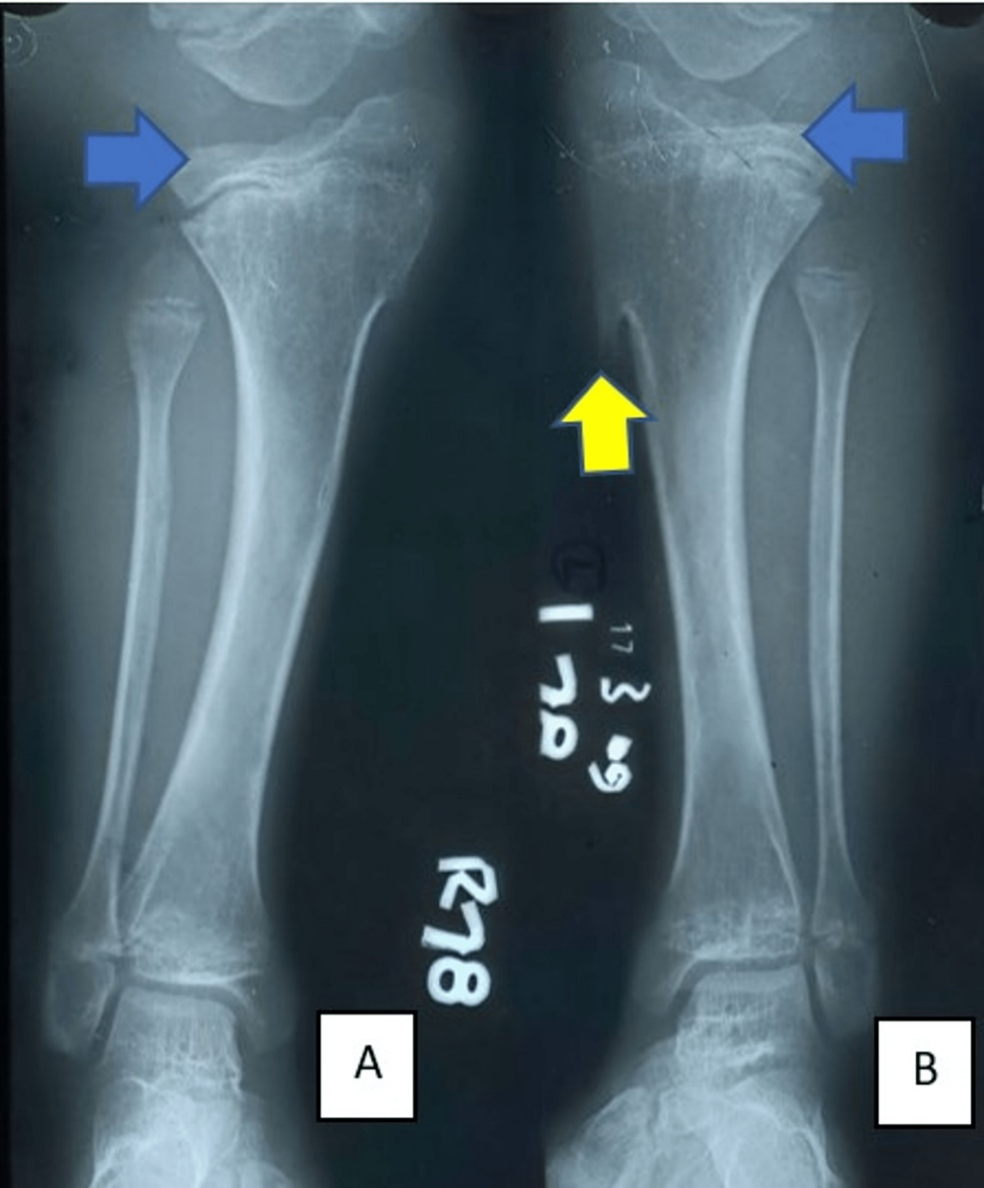
X-ray of bilateral knee with leg: A) dysplastic lateral tibial plateau of right knee (blue arrow). B) Dysplastic lateral tibial plateau of left knee (blue arrow) with exostosis in the proximal tibial region (yellow arrow).

**Figure 3:**
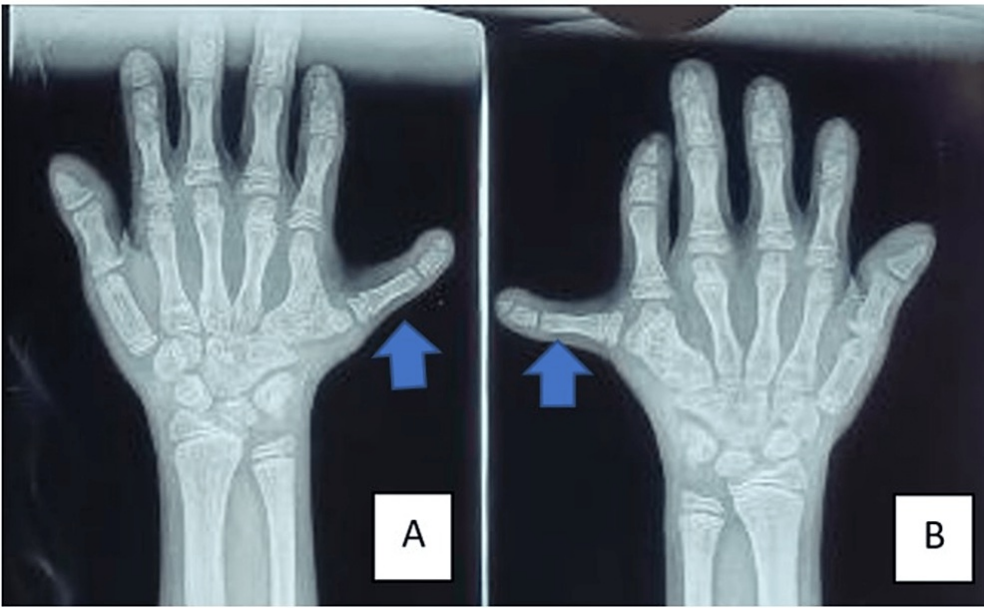
X-ray of bilateral hands: A) Polydactyly in the right hand (blue arrow). B) Polydactyly in the left hand (blue arrow).

**Figure 4:**
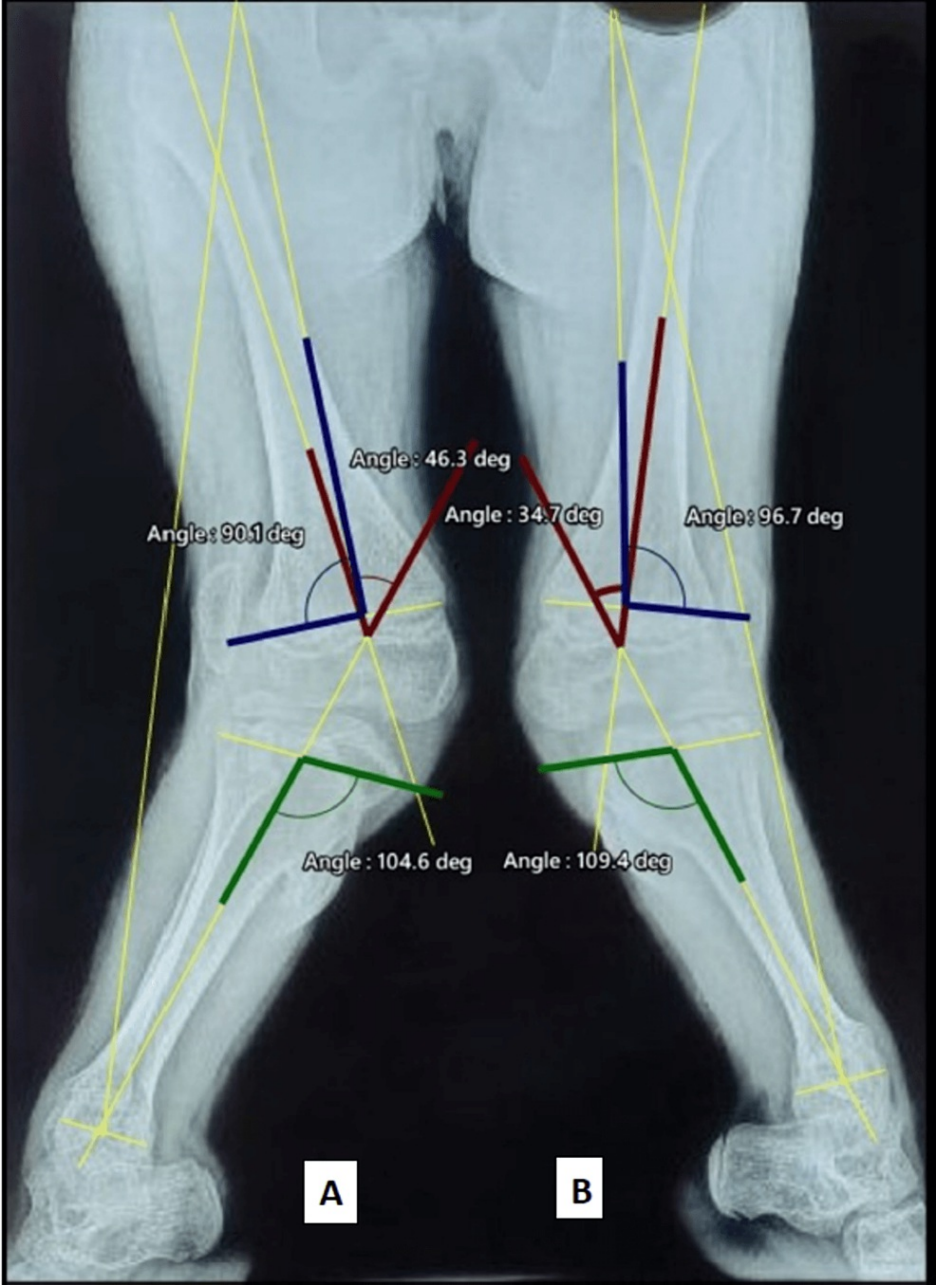
Radiological assessment measuring the tibio-femoral angle (red), lateral distal femoral angle (blue), medial proximal tibial angle (green) in right (A) and left (B) lower limbs.

Radiological investigations showed dysplastic proximal lateral tibial epiphysis (Figure 1) along with a proximal tibial exostosis (Figure 2). Radiographs of the wrist and hand showed post-axial polydactyly (Figure 3). Based on history, radiological evaluation, and clinical examination, a diagnosis of EVC was made. Since there were limited resources available and due to the financial constraints of the patient, a genetic evaluation was not done. As a part of the radiological assessment, the radiological angles were calculated (joint orientation angles and tibio-femoral angles) on both sides (Figure 4) (Table 1).

**Table 1:** Radiological assessment mentioning the angles in preoperative X-rays.

Angles (preop)	Right	Left
Tibio-femoral angle	46.3 degrees	34.7 degrees
Lateral distal femoral angle (LDFA)	90.1 degrees	96.7 degrees
Medial proximal tibial angle (MPTA)	104.6 degrees	109.4 degrees

Intervention

**Figure 5:**
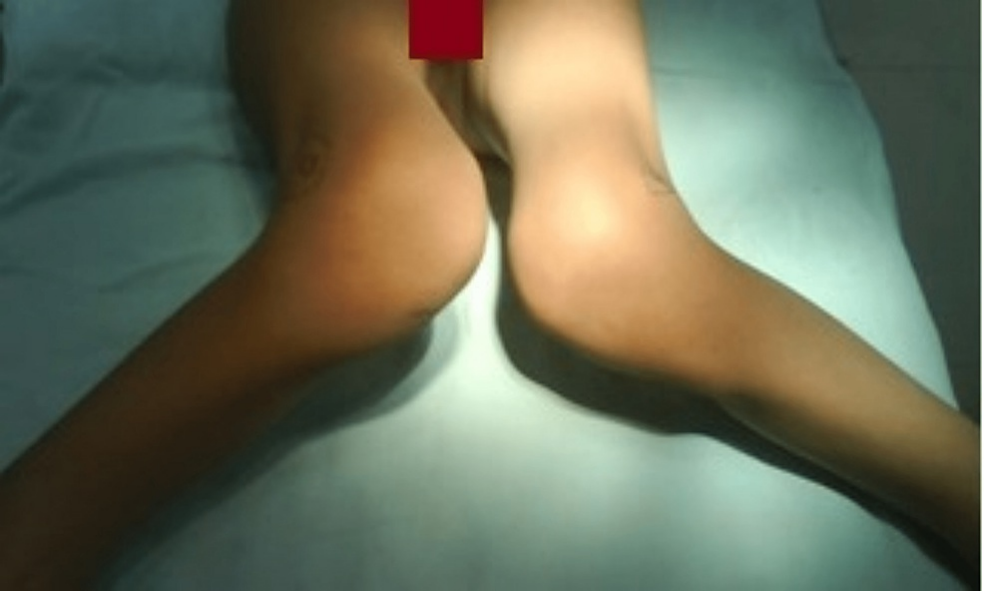
Persistent bilateral genu valgum deformity.

**Figure 6:**
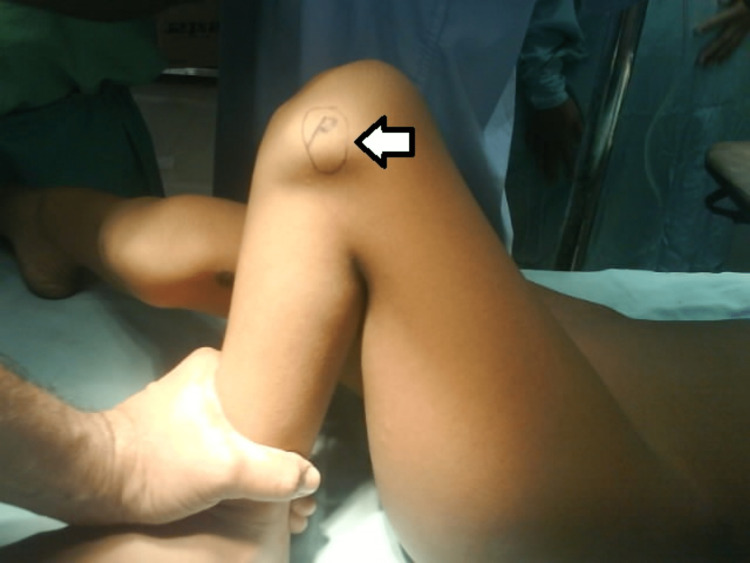
Laterally dislocated patella (white arrow).

The patient had already undergone at the age of 10 years a bilateral medial proximal tibia and distal femur epiphysiodesis with staples along with bilateral supracondylar femoral osteotomy at some other center. The correction following this surgery was not significant and the patellae were still dislocated and the patient still had difficulty walking (Figures 5, 6). The staples were removed when the patient had presented to our hospital one year later. 

Bilateral corrective osteotomy with soft tissue release followed by long leg casting and postoperative rehabilitative physiotherapy was planned. Since the procedure was extensive, it was done in a staged manner.

**Figure 7:**
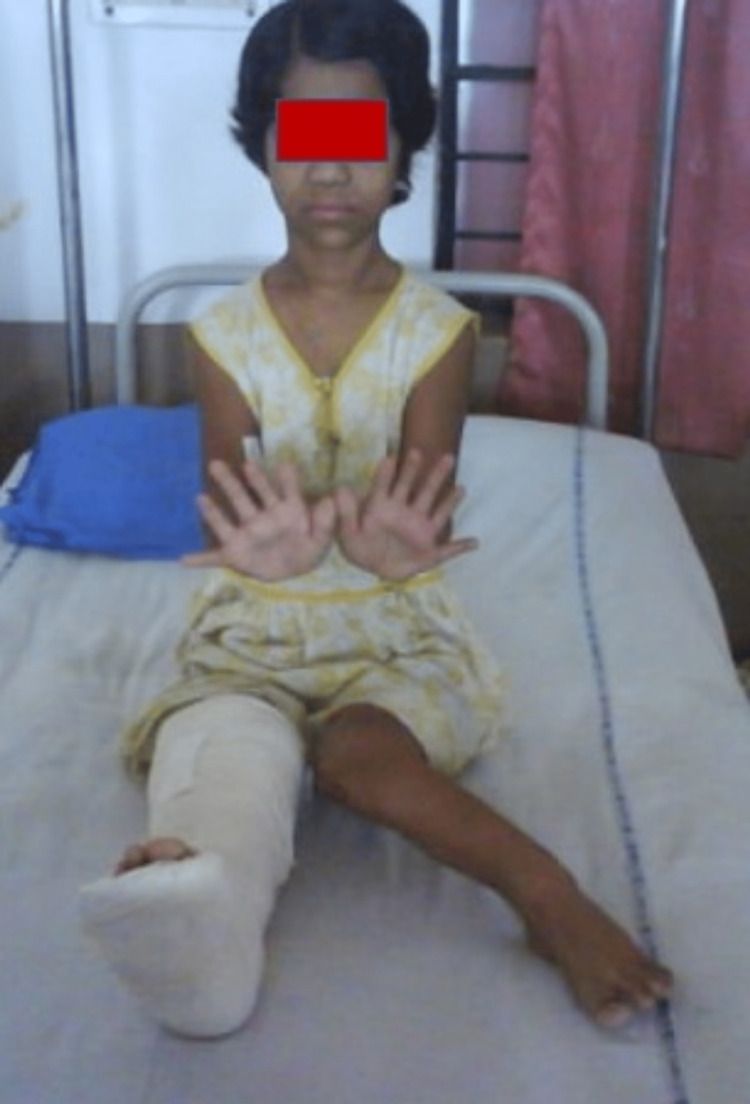
The procedure was planned in stages with the right side operated on first.

The radiographs were analyzed. The right lower limb was operated on first followed by the left limb 15 days later (Figure 7).

Surgical procedure

A bilateral corrective osteotomy was performed. The lateral release was performed including Z-plasty of the Ilio-tibial band, lateral retinacular release, quadriceps plasty, and decompression of the common peroneal nerve. 

On the medial side, medial retinacular plication, repair of medial patellofemoral ligament, was done to the medial border of the patella without any graft along with medial arthrotomy and the distal and lateral advancement of the vastus medialis obliquis. The lateral retinacular release was done. The medial capsule and retinaculum were exposed, and a proximal-based strip of the medial capsule was elevated and passed around the quadriceps tendon and sutured to the fascia over the adductor magnus tendon (Campbell procedure). Distal realignment by Roux-Goldthwaite procedure was also done. Medial closed wedge proximal tibial metaphysis osteotomy was performed and stabilized by k-wires. Flexion deformity was corrected with the Z-plasty of the Ilio-tibial band, Z-plasty of the hamstring tendon, and posterior capsulotomy. Complete correction of the deformity was achieved with a centrally located patella using these procedures.

The same procedure was repeated for the other limb.

Postoperative immobilization was done in a cast for six weeks, and weight-bearing started after radiological healing of osteotomy was seen.

Follow-up and outcomes

The patient was advised continuation of immobilization in a cast for one and a half months, following which the cast removal was done and rehabilitative physiotherapy was started.

The patient was followed weekly for the first two months to determine the outcome of physiotherapy and to modify physiotherapy if needed and then monthly for the next two years to check for relapses and later asked to follow up yearly.

By the end of six months of rehabilitation, she had a complete range of movements without any extension lag.

**Figure 8:**
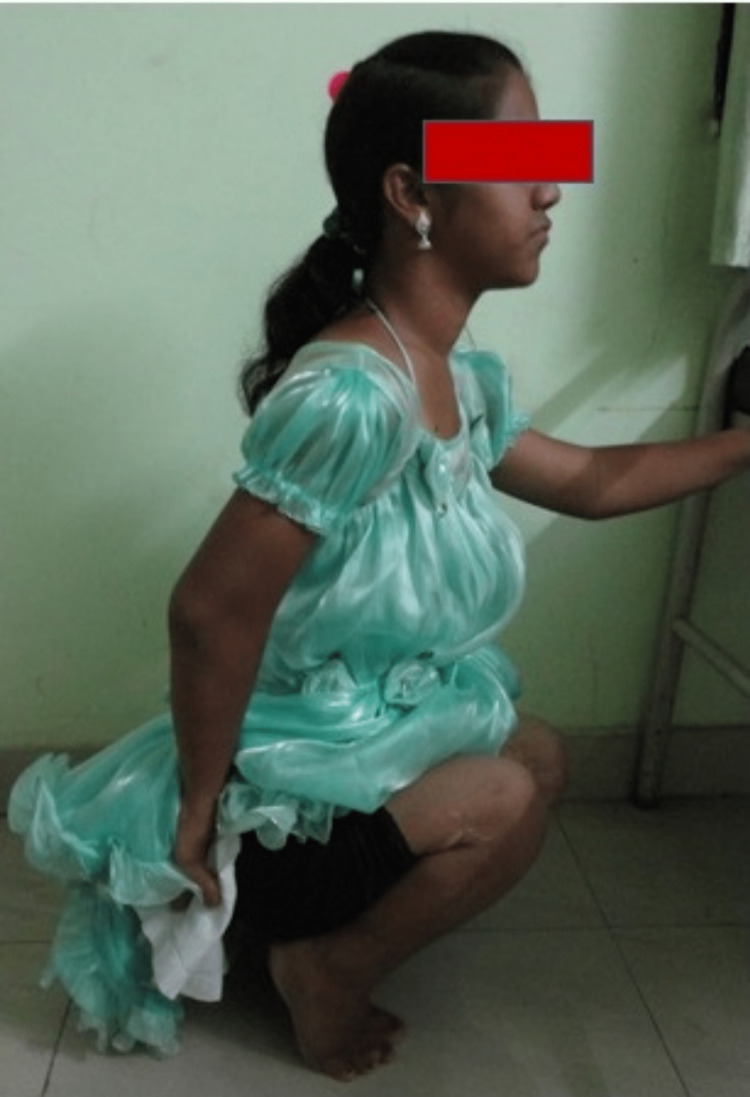
Patient able to squat at five-year follow-up.

**Figure 9:**
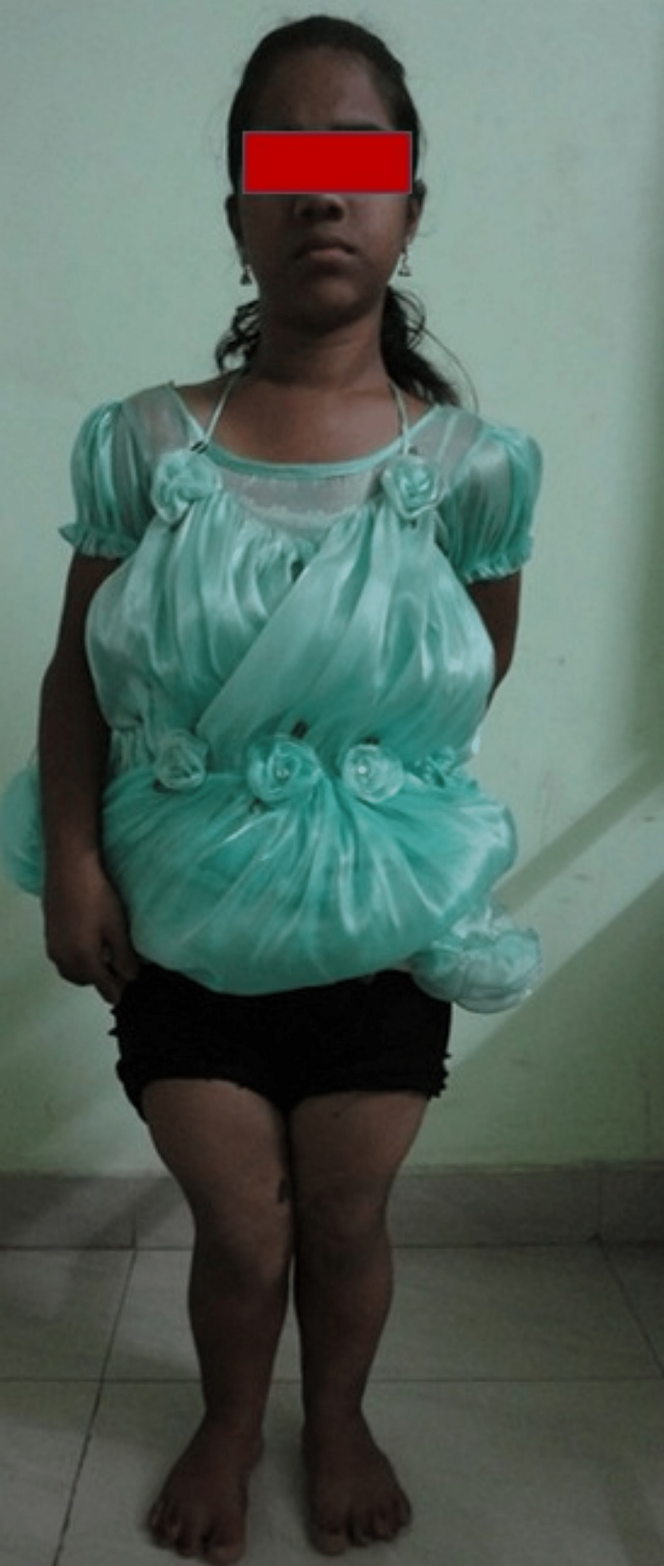
Significant deformity correction with no relapse at five-year follow-up.

**Figure 10:**
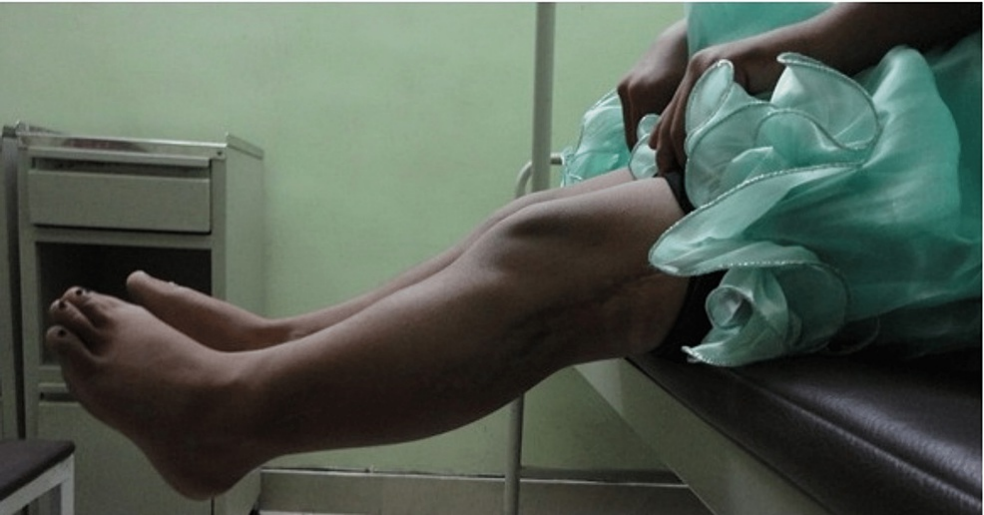
No extension lag post deformity correction.

**Figure 11:**
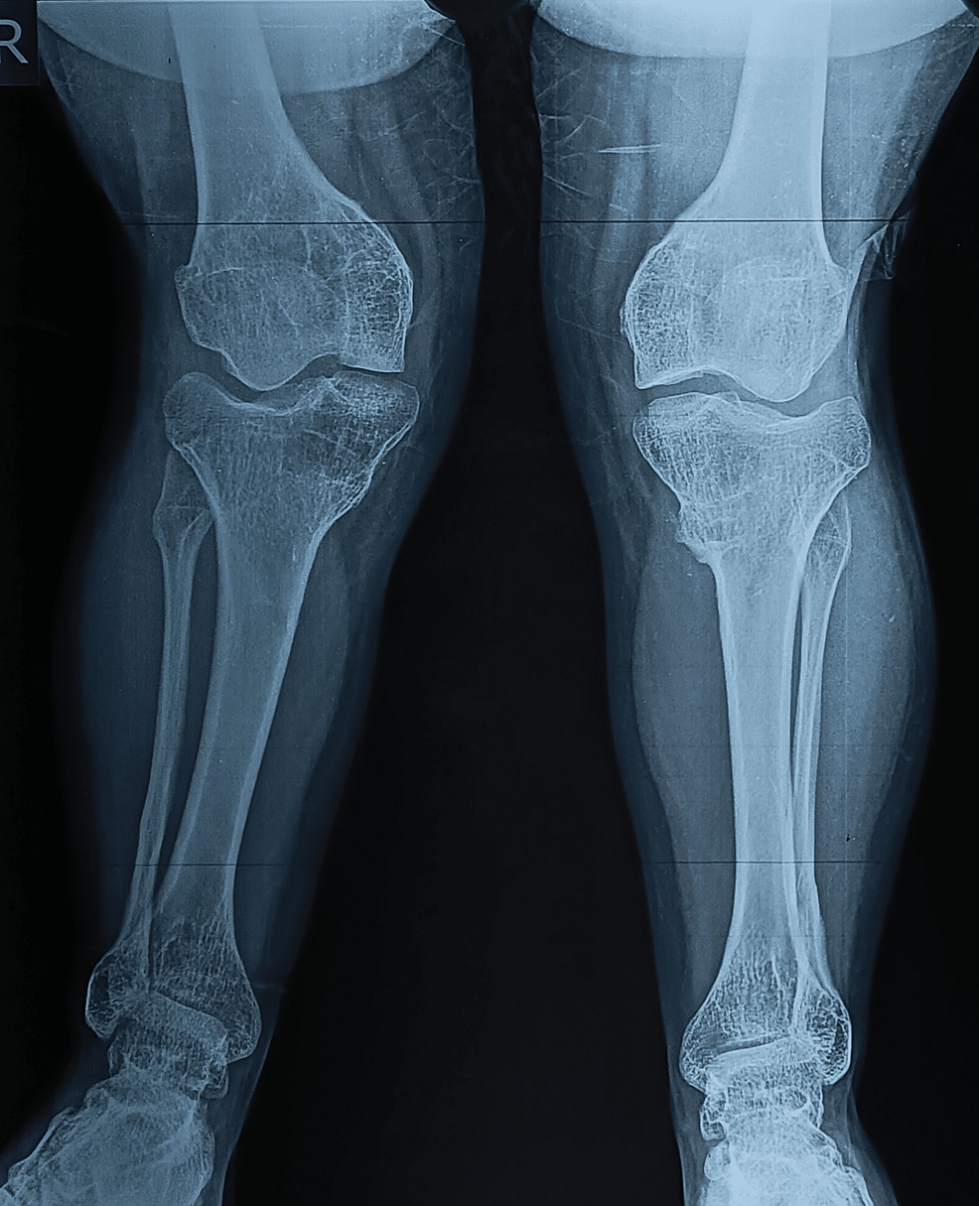
Radiographs at five-year follow-up showing good correction with no relapses.

At the follow-up of five years, at the age of 16 years, the correction was well maintained and both the patella were stable as was determined by weight-bearing X-rays and clinical examination. She did not have any instability of the knee and could sit and squat without difficulty. She was able to perform her daily activities without any problems (Figures 8-11).

**Figure 12:**
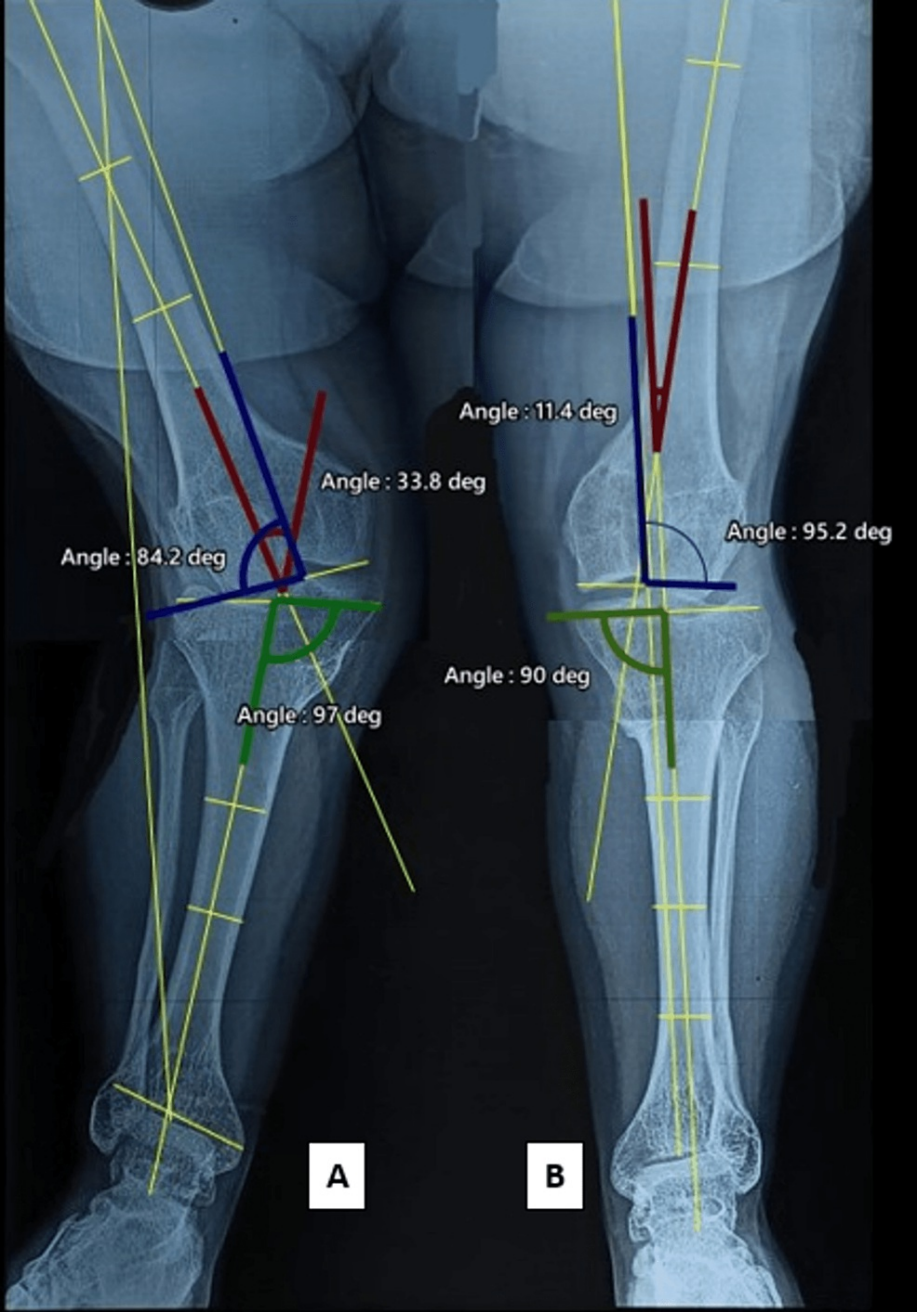
Radiological assessment at five-year follow-up measuring the tibio-femoral angle (red), lateral distal femoral angle (blue), medial proximal tibial angle (green) in right (A) and left (B) lower limb.

Despite the extensive nature of the disease and prolonged immobilization in lower leg casts, the patient did not have any limb length discrepancy or land up in any kind of postoperative complications. The patient had 0-150 degrees of flexion as evidenced by the follow-up clinical photo at the five-year follow-up. On follow-up at five years the radiological angles were calculated and a significant correction was observed which was persistent at the follow-up (Figure 12) (Table 2).

**Table 2:** Radiological assessment mentioning the angles in postoperative X-rays.

Angles (postop)	Right	Left
Tibio-femoral angle	33.8 degrees	11.4 degrees
Lateral distal femoral angle (LDFA)	84.2 degrees	95.2 degrees
Medial proximal tibial angle (MPTA)	97 degrees	90 degrees

## Discussion

EVC syndrome is an uncommon condition with an autosomal recessive inheritance. The patients present with a varied range of findings, especially grave valgus deformity in the knee joint. The genu valgum deformity is one of the gravest angular deformities in orthopedics. It is due to the involvement of the lateral tibial plateau, which is affected by the primary genetic-based dysplasia and profound soft tissue contracture that forces the shaft of the tibia into valgus deformity [3]. Proximal to the distal cortical thickness and short segments of tubular bones are the radiological diagnostic characteristics. The bones of the forearm and lower extremities are characterized by chondrodystrophy [3].

Surgical treatment is often required for the management of dislocation of the patella and valgus knee deformity. Osteotomy, epiphysiodesis, and Ilizarov fixator are among various treatment modalities for correction of valgus malalignment and hence to improve functional outcomes like gait. However, in EVC syndrome patients, there is a frequent recurrence because of lateral tibial plateau depression [4-6]

We performed bilateral tibial and femoral osteotomy with soft tissue release. Fraser and Metrakos in 1954 [7] suggested osteotomies of the supracondylar femur to treat genu valgum deformities. A similar procedure was reported by Pinelli in 1990 [4], where he stated that patients with EVC syndrome should be treated with medial epiphysiodesis as there are frequent recurrences with his technique. These techniques provided poor results and recurrence, which was not seen in our study. Epiphysiodesis by use of staples was performed by Courivisier in 2009 [8] who found it to be a safe alternative method to achieve the correction. Similar procedures were performed by Frantz in 1971 [9], Zuege et al. in 1979 [10], and Fraser et al. in 1995 [11]. The procedure was previously avoided in pediatric age group patients due to fear of permanent physeal closure as was believed by various authors. Our patient was previously operated on with a similar treatment modality but the result of the correction was not significant.

Martin Gottliebsen et al in 2013 [12] performed a randomized control trial on 26 patients to correct the genu valgum deformity using plating and staples and found good results with similar time of treatment for both the modalities and found recurrence associated with the use of the plate. Ballal et al in 2010 [13], in 25 consecutive children with a follow-up of 12.4 months who were treated by flexible two-hole titanium eight plates, found rebound deformity in one patient. Milgram et al. in 1975 [5] performed osteotomy of both tibiae and fibulae for correction of the valgus deformity. Milgram did not perform any soft tissue procedure for the correction of deformity and reported recurrence. In our present study, we performed an osteotomy of the tibia with soft tissue release and reported no recurrence. Shibata et al. in 1999 [6] treated bilateral genu valgum deformity with tibia and fibula osteotomy with soft tissue release and reported recurrence and under-correction.

Fukuda et al. in 2012 [14] performed bilateral dome osteotomies with k-wire for genu valgum deformity. On follow-up, the patient was re-operated with varus close wedge osteotomy and soft tissue release to correct the recurrence. Eylon et al. in 2008 [15] in his study mentioned the results of high tibial osteotomy combined with soft tissue release in three knees with genu valgum deformity in patients with EVC syndrome and stated that in a high tibial osteotomy, the recurrence is likely as the pathology is not addressed and soft tissue release is necessary. Jöckel et al. in 2012 [16] performed osteotomies for genu valgum deformities and had to perform multiple surgeries due to repeated recurrences and hence concluded that soft tissue release is necessary for these kinds of recalcitrant deformities and adequate osteotomy despite being performed alone is not often enough. In 2007, Morsy et al. [17] mentioned Ilizarov external fixator as a treatment modality for bilateral valgus deformity in a girl with EVC syndrome aged 13 years. They reported the patient to have a normal gait and mechanical axis and had relief from pain.

Intra-articular osteotomy as a treatment modality for genu valgum has been mentioned by Kamada et al. in 2017 [18], Paley and Tetsworth in 1991 [19], and Feldman et al in 2016 [20]. Paley and Tetsworth and Feldman et al. mentioned excellent clinical outcomes in their patients. Kamada et al. stated that for severe valgus deformity in EVC patients, improvement of joint congruity can be done by elevation of the anterolateral aspect of the tibia by an intra-articular osteotomy. These studies have provided the closest results to our present study in terms of postoperative outcome and recurrence.

In our case, the patient had previously undergone bilateral supra-condylar femur osteotomies and distal femoral and proximal tibial epiphysiodesis with staples without any soft tissue procedures but the correction was not satisfactory, highlighting the importance of soft tissue procedures for correction.

On presentation, planning was done after studying the scanograms and combined soft tissue release procedures with osteotomies were done. The patient had a good outcome postoperatively.

There have been cases of knee stiffness that can be treated by knee manipulations. These manipulations might lead to a fracture of the distal femur. Due to the need for extensive dissection, there is a chance of foot drop due to injury of the common peroneal nerve. Chances of non-union are there, which can be managed with revision surgery. Due to extensive dissection, complications such as multiple surgeries, flap necrosis, and infection with the potential of significant blood loss can occur.

No such complications occurred in our current case.

## Conclusions

Due to the rare nature of the deformity, the studies on the topic are limited with limited information on the surgical management and outcome. The severity of the deformity and the relapsing nature of the deformity pose a stern challenge to the surgeons. Osteotomy with soft tissue release is necessary to achieve good correction and prevent relapses. Surgical management though complex can achieve good results with good postoperative function if proper preoperative evaluation and planning are done.
